# Psilocybin-assisted therapy and HIV-related shame

**DOI:** 10.1038/s41598-024-68908-4

**Published:** 2024-08-02

**Authors:** Nicky J. Mehtani, Mallory O. Johnson, Peter S. Hendricks, Jennifer Mitchell, Brian T. Anderson

**Affiliations:** 1 Department of Psychiatry and Behavioral Sciences, University of California, San Francisco, Sandler Neurosciences Bldg., Rm 510, 675 Nelson Rising Lane, San Francisco, CA 94143 USA; 2 Department of Medicine, Division of Prevention Science, University of California, San Francisco, San Francisco, CA USA; 3 Department of Psychiatry and Behavioral Neurobiology, University of Alabama at Birmingham, Birmingham, AL USA; 4 Department of Neurology, University of California, San Francisco, San Francisco, CA USA; 5 Department of Medicine, Division of General Internal Medicine, University of California, San Francisco, San Francisco, CA USA

**Keywords:** Psilocybin, Shame, HIV, Psychotherapy, Psychedelics, Stigma, LGBTQ, Psychology, Emotion, Stress and resilience, HIV infections, Addiction

## Abstract

As a proposed mediator between stigma-related stressors and negative mental health outcomes, HIV-related shame has been predictive of increased rates of substance use and difficulties adhering to antiretroviral treatment among people with HIV. These downstream manifestations have ultimately impeded progress toward national goals to *End the HIV Epidemic*, in part due to limited success of conventional psychotherapies in addressing HIV-related shame. In a pilot clinical trial (N = 12), receipt of psilocybin-assisted group therapy was associated with a large pre-post decrease in HIV-related shame as measured by the HIV and Abuse Related Shame Inventory, with a median (IQR) change of − 5.5 (− 6.5, − 3.5) points from baseline to 3-months follow-up (*Z* = − 2.6, *p* = 0.009, r = − 0.75). A paradoxical exacerbation of sexual abuse-related shame experienced by two participants following receipt of psilocybin raises critical questions regarding the use of psilocybin therapy among patients with trauma. These preliminary findings carry potential significance for the future of HIV care.

## Introduction

While extraordinary advancements in the efficacy and accessibility of antiretroviral therapy (ART) over the past three decades have transformed HIV into a manageable, chronic medical condition, people with HIV (PWH) in the U.S. continue to experience disproportionately high rates of psychological distress and substance use disorders (SUDs)^1,2^. The challenges posed by these mental health co-morbidities have been widely recognized as among the greatest barriers toward achieving the visionary goal of *Ending the HIV Epidemic*^1^.

Shame—defined as an intensely painful emotion resulting from negative self-evaluation following a perceived deviation from a social or moral code—is often accompanied by feelings of inferiority, defectiveness, self-consciousness, and isolation, and is thought to play a significant role in mediating the relationship between stigma-related environmental stressors and negative mental and physical health outcomes among PWH^3–6^. The nature of shame is further complicated for individuals who have experienced sexual abuse or identify as sexual and gender minorities (SGM), who account for an estimated 30–50% and 70% of U.S. PWH, respectively^4,7^. Shame related to HIV and sexual abuse has been associated with increased rates of depression, anxiety, post-traumatic stress disorder (PTSD), homelessness, and incarceration^4,5^. HIV-related shame is also predictive of poor coping strategies, including substance use and risky sexual behaviors—activities that may temporarily alleviate shame but ultimately perpetuate it while contributing to reduced health-related quality of life, increased risk for non-adherence to ART, and continued HIV transmission within vulnerable communities^5,6^. Feelings of shame are also known to increase cortisol and proinflammatory cytokine activity, predisposing minoritized populations to increased risks of poor physical health outcomes, including cardiovascular and metabolic diseases^8^.

In contrast to the related constructs of societal HIV stigma and discrimination, which are externally mediated and often necessitate structural changes or widespread advocacy to address effectively, shame is an internally experienced emotional response that can be potentially altered through individual-level behavioral interventions^4^. However, shame operates at a deep and sometimes subconscious psychological level—one that is generally more engrained than the cognitive concept of guilt^3^. Alleviating shame entails deconstructing the subtle ways in which negative self-conception can become subliminally encoded into one’s perception of the world^3^. While different psychotherapies have emerged as vital components of clinical care for PWH and may help reduce shame, effective forms of psychotherapy are generally time-consuming, demand a high degree of motivation, and may pose unique challenges for individuals suffering from shame, which often serves as a barrier toward care engagement^3–5^.

In recent years, early phase clinical trials have demonstrated the preliminary efficacy of psychedelic therapies in addressing various forms of psychological distress, including PTSD, SUDs, and, among patients with serious medical illnesses, despair^9,10^. Psychedelic therapies combine conventional forms of psychotherapy with powerful psychoactive substances, such as psilocybin and 3,4-methylenedioxymethamphetamine (MDMA), which may produce temporary alterations in a person’s perception, mood, and/or cognitive processes^9,10^. Although no studies have formally evaluated the role of psychedelics in addressing shame, psychotherapy interventions involving psilocybin have been hypothesized to offer enduring mental health benefits to SGM individuals by disrupting self-perpetuating “shame spirals” that are experienced disproportionately by this population^11^.

The current study investigated changes in HIV- and sexual abuse-related shame experienced among gay-identified, older, long-term AIDS survivors participating in the only clinical trial to-date that has explicitly examined the effects of psychedelics in SGM patients^12^. Treatment involved four preparatory group therapy sessions, a single mid-treatment individual psilocybin session (open-label and dosed orally at 0.30–0.36 mg/kg), and four-to-six integrative group therapy sessions all administered over a 6-week period^12^. Exploratory analyses aimed to evaluate whether psilocybin-assisted psychotherapy reduced HIV- and sexual abuse-related shame from baseline to 3-months follow up.

## Results

Complete pre and post data were collected for all n = 12 study participants administered the HIV and Abuse Related Shame Inventory (HARSI)^4^ (Table 1). Cronbach's α for the HIV- and sexual-abuse related shame subscales in this sample were 0.96 and 0.94, respectively, indicating excellent internal reliability.

**Table 1:** Baseline characteristics of psilocybin group therapy study participants (N = 12) administered the HIV and Abuse Related Shame Inventory (2017–19).

Demographics	n (%)
Age (years), mean (SD)	58.7 (4.6)
Male sex and gender, n (%)	12 (100%)
Race/ethnicity, n (%)	
White	9 (75%)
Multiracial	3 (25%)
Hispanic	2 (17%)
Annual income (U.S. dollars), n (%)	
< $20,000	3 (25%)
$20,000–$49,999	5 (42%)
$50,000–$99,999	2 (17%)
≥ $100,000	2 (17%)
Education, n (%)	
Some college	3 (25%)
College degree	4 (33%)
Some graduate school	2 (17%)
Graduate degree	3 (25%)
Marital status, n (%)	
Single	5 (42%)
Married or partnered	6 (50%)
Separated or divorced	1 (8%)
Prior history of classic psychedelic use*, n (%)	11 (92%)

*Participants reported a median (IQR) of 8 (3,21) prior experiences using a classic psychedelic (i.e., psilocybin, lysergic acid diethylamide, *N*,*N*-dimethyltryptamine or mescaline), with a median (IQR) of 6 (1,28) years since their last use.

**Figure 1:**
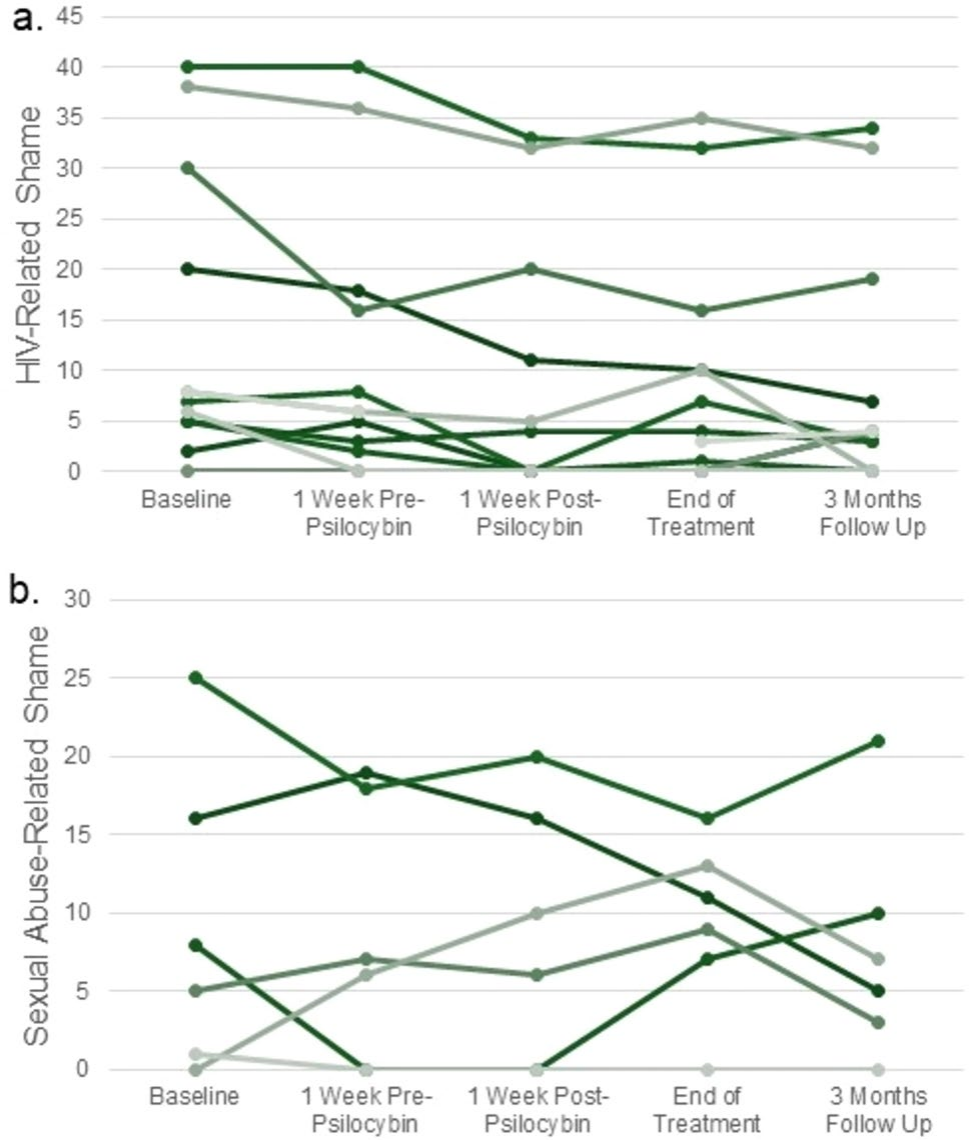
Participant scores on the HIV and Abuse Related Shame Inventory at multiple time points from baseline to three months following a 6-week psilocybin-assisted group therapy intervention (2017–2019). (**a**) HIV-related shame scores declined from baseline to all time points following receipt of psilocybin (*p* < 0.05; n = 12). (**b**) More variable trajectories were observed among sexual abuse-related shame scores over time (n = 6).

A Friedman test indicated significant differences on scores of both HARSI sub-scales across five time points from baseline to 3-months following psilocybin dosing (χ^2^(2) = 45.25, *p* < 0.001 for HIV-related shame; χ^2^(2) = 16.66, *p* = 0.005 for sexual abuse-related shame). Pairwise comparisons evaluated using the Wilcoxon matched-pairs signed-rank test indicated significant reductions in HIV-related shame from baseline to all time points following but not prior to psilocybin dosing (1-week pre-psilocybin: Z = − 1.9, *p* = 0.056, r = − 0.60; 1-week post-psilocybin: Z = − 2.9, *p* = 0.004, r =− 0.87; 3-weeks post-psilocybin: Z = − 2.5, *p* = 0.012, r = − 0.79; 3-months post-psilocybin: Z = − 2.6, *p* = 0.009, r = − 0.75). There was an overall median (IQR) change of − 5.5 (− 6.5, − 3.5) points in HIV-related shame from baseline to 3-months follow up (Fig. 1a). Pairwise comparisons indicated no overall change in sexual abuse-related shame from baseline to any of the pre- or post-psilocybin time points among the subset of participants reporting a history of sexual abuse (n = 6), and two participants experienced increases in sexual abuse-related shame following psilocybin-assisted group therapy (Fig. 1b).

## Discussion

Findings from this study provide insights into the potential of psilocybin-assisted psychotherapy as an innovative approach for addressing shame among PWH. Despite a small sample size, results revealed a statistically significant reduction in HIV-related shame among older, long-term AIDS survivor men following completion of a 6-week psilocybin-assisted group therapy intervention that remained durable at 3 months follow-up with a large effect size (r = − 0.75). While pre-post changes in sexual abuse-related shame sub-scores were not significant, it is noteworthy that half of the 12 study participants reported a history of sexual abuse and that two experienced an exacerbation of sexual abuse-related shame following receipt of psilocybin (Fig. 1b). These preliminary findings support the pursuit of further research into the use of psychedelic therapies to address shame and associated mental health sequelae among PWH.

The observed overall reduction in HIV-related shame following psilocybin-assisted group therapy is a promising signal of potential benefit. Of note, the median (IQR) baseline score among participants in this study of 7.5 (5, 22.5) was lower than the mean (SD) scores reported in larger population-based studies, which have ranged from 17.1 (13.4) to 17.8 (13.1) out of 52^4,13^. In our sample, reductions in HIV-related shame appeared greatest among participants with baseline scores ≥ 20 (Fig. 1a). While previous research has demonstrated the efficacy of psychedelic therapies in addressing various forms of psychological distress, including depression, anxiety, and SUDs^9,10^, this is the first study to empirically evaluate the impact of a psychedelic therapy on shame. These findings also draw from the only interventional trial to-date focused explicitly on evaluating the effects of psilocybin therapy among SGM patients and PWH^12^, who face unique challenges in accessing and maintaining care engagement^3–6^. While all participants in this trial were virologically suppressed and taking ART, for many PWH, the impacts of shame-mediated mental health conditions contribute toward challenges with ART adherence^5^. Therefore, by offering a promising approach toward addressing HIV-related shame, psilocybin therapy may indirectly support improved adherence to HIV pharmacotherapies, subsequent improvement in clinical outcomes, and reductions in HIV transmission.

Importantly, the trial’s behavioral intervention involved a form of group therapy, which was modeled on brief Supportive Expressive Group Therapy^14^ and its prior adaptations for PWH^15,16^. Compared with individual therapy, group therapy itself may foster decreased shame among PWH^16^. While not statistically significant, our analyses revealed a large decrease in HIV-related shame from baseline to 1-week pre-psilocybin dosing (r = − 0.60, Fig. 1a), suggesting that the group therapy alone likely contributed to decreased HIV-related shame among participants. However, further decreases were experienced following psilocybin dosing. Therefore, while the contributory effects of group therapy and psilocybin in this study cannot be disentangled, overall findings suggest synergistic effects of these modalities on reducing HIV-related shame.

These data also provide preliminary empiric support for a recently proposed theory suggesting a unique therapeutic potential of psychedelics in alleviating chronic shame among SGM^11^. This theory aligns with the Self-entropic Broadening framework, which posits that psychedelics induce long-term mental health improvements by reducing self-focus and promoting hyper-associative thinking, facilitating a broadening of attentional scope and an expansion of thought-action repertoire^11^. In the context of this study, chronic shame related to HIV could be considered a form of negative self-focused attention, and psilocybin may mitigate the physiological responses associated with this shame^11^. Such a reduction in shame may encourage PWH and SGM to adopt a wider range of adaptive coping strategies to regulate negative affect. This might involve engaging in mental health care or other health-promoting activities instead of seeking escape through substance use or risky sexual behaviors, thus allowing for a potential break from activities that could perpetuate a ‘spiral’ of shame^11^.

The unexpected increase in sexual abuse-related shame observed in two participants raises critical questions regarding the use of psilocybin therapy in populations known to have histories of trauma. While two phase III clinical trials have demonstrated efficacy of MDMA-assisted therapy in treating PTSD^17,18^ and studies of psilocybin therapy for PTSD are currently underway, existing literature on the use of psilocybin in PTSD is limited^19^. In the current analysis, one participant reported no sexual abuse-related shame at baseline, yet his sub-score increased to 13 by the end-of-treatment (Fig. 1b). Based on clinical observation, he demonstrated increased comfort with self-disclosure in group settings during the first two weeks following psilocybin but subsequently experienced an exacerbation of social anxiety and self-critical thinking in the therapy group. While future research is needed to interrogate these temporal relations, given that his sub-score decreased from 13 to seven by 3-months follow-up, his experience may ultimately reflect habituation to distressing cognitions previously avoided^20^. Nevertheless, these findings underscore the importance of addressing ethical considerations, safety, and potential adverse effects of psychedelics among diverse populations as this field of research evolves. For example, specific counseling regarding psilocybin-related risks may be warranted for people with significant histories of trauma, as some symptoms might be expected to temporarily worsen before improving. Future trials should also consider potential needs for extended integration therapy sessions or inclusion of multiple psychedelic dosing sessions, which may provide participants with multiple opportunities to address complex trauma.

This study has important limitations. The absence of a control group and small sample size restrict the interpretability of results, as the original trial was not designed or powered to evaluate efficacy outcomes. Additionally, the trial intentionally selected for a relatively homogenous group of gay-identified, cis-gender, older-adult male participants to enhance a sense of trust and safety within group therapy cohorts over a brief period. These inclusion criteria notably narrow the generalizability of study findings. Moreover, PWH from a single urban area who volunteered to participate in a trial of psilocybin group therapy may not be representative of the broader population of PWH. In particular, the over-representation of white men in this sample (75%) is a notable shortcoming given the extent to which sexual and minority of men of color have been disproportionately affected by the HIV epidemic. Finally, the use of self-reported survey data is susceptible to recall and social desirability bias. Nevertheless, the HARSI has exhibited excellent internal consistency and test–retest reliability in other populations^4,13^, and its use of a 1-week recall period should help minimize such biases.

In future clinical trials of psychedelic therapies, it will be critical to incorporate measures of shame and recruit larger, more diverse populations, including PWH across various age groups, lengths of time since HIV diagnosis, sexual and gender identities, substance use patterns/diagnoses, and sociodemographic and racial/ethnic backgrounds, with interventions tailored to the specific needs of these communities. Long-term follow-up studies may provide insights into the dynamics of HIV- and sexual abuse-related shame following psilocybin therapy and their impact on mental and physical health outcomes. Moreover, the incorporation of complementary measures of self-efficacy and resilience and the expansion of qualitative research may further elucidate the psychological mechanisms underlying the observed changes.

## Conclusions

Findings from this study demonstrate promise for the potential of psilocybin therapy in addressing shame, a pervasive issue among PWH that is associated with disparities in mental and physical health outcomes. While further research is needed, this analysis instills hope for the development of innovative and accessible psychedelic therapy interventions that may alleviate the burden of shame experienced by PWH.

## Methods

### Study design and population

This study utilized data from an open-label pilot trial of psilocybin-assisted group therapy for the treatment of moderate-to-severe demoralization in older long-term AIDS survivor men conducted in San Francisco, California (USA) between July 2017 and January 2019 (NCT02950467). The trial included three group therapy cohorts, each consisting of six participants (N = 18). The study was approved by the UCSF Institutional Review Board (CHR#15-17825), and the main outcomes have been published separately^12^. Written informed consent was obtained from all subjects, and all methods were carried out in accordance with relevant guidelines, regulations, and Good Clinical Practices.

### Measures

The HIV and Abuse Related Shame Inventory (HARSI)^3^ was administered to study participants in the last two group therapy cohorts (n = 12) at baseline, 1 week pre- and post-psilocybin dosing, end of treatment, and 3-months follow-up. The 13-item HIV-related shame subscale, which reflects multiple aspects of past-week HIV-related shame, was administered to all participants, and the 9-item Sexual abuse-related shame subscale was administered to participants who reported any lifetime history of sexual abuse. All responses were rated on a 5-point scale from 0 (not at all) to 4 (very much) and summed to create a total possible score of 52 for HIV-related shame and 36 for Sexual abuse-related shame^4^. The HARSI has been validated and found to have high internal reliability among PWH with a history of sexual abuse^3^ as well as PWH aged ≥ 50 years^13^.

### Statistical analysis

Internal reliability estimates of the two HARSI subscales were calculated based on data from this study sample using Cronbach’s α. A Friedman test at α = 0.05 was conducted to initially evaluate whether there were significant differences in each of the shame sub-scale scores across the different time points. If the Friedman test indicated a significant result, post-hoc pairwise comparisons were conducted using the Wilcoxon matched-pairs signed-rank test to evaluate whether baseline measures were significantly different from those observed 1-week pre-, 1-week post-, 3-weeks post-, and 3-months post-psilocybin dosing. Subscale scores were plotted over the duration of the 6-week study intervention to support interpretation of potential effects from group therapy and psilocybin dosing sessions. Given the small sample size and exploratory goals of this analysis, the nested nature of data from two separate group therapy cohorts was not accounted for, and no corrections were made for multiple comparisons. Analyses were conducted in Excel and STATA, version 18.0 (Stata Corp., College Station, TX, USA).

## Data Availability

The dataset analyzed during the current study is not publicly available to protect the privacy of study participants given the small size of the recruited sample. However, aggregated data is available online (https://classic.clinicaltrials.gov/ct2/show/results/NCT02950467) and de-identified individual data may be available from the corresponding author upon reasonable request.
